# Determination of the Contents of Antioxidants and Their Degradation Products in Sodium Chloride Injection for Blood Transfusion

**DOI:** 10.1155/2020/8869576

**Published:** 2020-06-22

**Authors:** Bo Tao, Gang Wang, Zongning Yin, Xiaocong Pu, Yan Jiang, Luohong Zhang, Jie Cheng, Yong Li, Jiayu Zhang

**Affiliations:** ^1^Key Laboratory of Drug Targeting and Drug Delivery Systems, West China School of Pharmacy, Sichuan University, No. 17, Block 3, Southern Renmin Road, Chengdu 610041, China; ^2^Sichuan Institute for Food and Drug Control, Chengdu 611731, China; ^3^Sichuan Taipingyang Pharmaceutical Co., Ltd., Chengdu 611731, China

## Abstract

The infusion bag is mainly made up of polyolefin polymer. Antioxidants are usually added to these polymer materials in the production process to prevent the materials from aging and enhance the stability of the materials. Because of the potential harm of antioxidants to human body, it is necessary to limit the amount of antioxidants migrating to the pharmaceutical solutions. In the present study, we developed and validated the HPLC method for the simultaneous quantification of antioxidants and their degradation products migrating to sodium chloride solution for injection. A total of six antioxidants and six their degradation products were separated and simultaneously determined by using a Waters Symmetry RP18 column (250 × 4.6 mm, 5 *μ*m) and gradient elution of methanol/acetonitrile/acetic acid-water (1 : 99, v/v) at a flow rate of 1.0 mL/min. The detective wavelength was set at 277 nm, and the column temperature was maintained at 35°C. The method was validated in terms of limit of detection (LOD, 0.011–0.151 *μ*g/mL), limit of quantification (LOQ, 0.031–0.393 *μ*g/mL), intraday precision (0.25%–3.17%), interday precision (0.47%–3.48%), linearity (0.1–46.8 *μ*g/mL, *r* > 0.9994), stability (0.35%–3.29%), and accuracy (80.39%–104.31%). In the extraction experiment, antioxidants, BHT, 1010, 1330, 1076, and 168, and their degradation products, 1310 and DBP, were detected in the packaging materials. Only 1310 was detected in the migration experiment. The maximum daily dosage of sodium chloride for blood transfusion is three bags, and the content of 1310 in long-term testing samples is from 0 to 12 months ranging from 37.44 *μ*g/3 bags to 48.71 *μ*g/3 bags. The daily intake of 1310 did not exceed 48.71 *μ*g, which was much lower than its permitted daily exposure (PDE, 300 *μ*g/day). Therefore, the antioxidants and their degradation products migrating into the drug solution would not cause drug safety risks.

## 1. Introduction

The earliest intravenous infusion container is the feather needle tube and animal bladder invented by Christopher and Robert [1]. Glass bottles have been used as infusion containers for nearly a century since it was invented in 1920. The glass bottle has the advantages of higher transparency, excellent pressure resistance, and good thermal stability. However, the glass bottle packaging is poorly sealed, easy to peel off, fragile [2, 3], and not conducive to transportation and has invisible cracks caused by collision and only semiopen infusion mode can be used [4]; the rubber plug must come in contact with the liquid medicine directly. All of these factors may cause the contamination of the drug solution. In recent years, glass bottles have gradually been replaced by non-PVC multilayer coextrusion film infusion soft bags [5].

The 0.9% sodium chloride injection for blood transfusion is used together with the Baxter Fenwal CS-3000 plus blood component separator and a single disposable blood cell separator. About 200 mL sodium chloride injection is used for flushing the air in the pipeline of single disposable blood cell separator before blood collection. After blood collection, about 300 mL sodium chloride injection is used for flushing the residual blood components in the pipeline of single disposable blood cell separator back to the body. Therefore, the dosage of sodium chloride injection is about 500–600 mL each time. The maximum daily dose of sodium chloride injection for blood transfusion is about three bags (250 mL/bags).

Sodium chloride injection for blood transfusion enters the human body by intravenous injection. Therefore, the safety of packaging materials for transfusion is very important. At present, the widely used packaging materials for infusion are non-PVC multilayer coextrusion films prepared by mixing polypropylene and polystyrene-ethylene-butylene-styrene block copolymer (SEBS) with different proportions.

The infusion bag and the packaged product will go through a series of long processes, such as packaging, sterilization, transportation, and shelf display. During processing, storage, and use, they will age due to the influence of light, heat, air, and other factors. The automatic oxidation of polymer is a process consisting of a series of free radical reactions. Adding antioxidants to the polymer can prevent the material from aging, property change, damage, yellowing, and embrittlement, leading to an enhanced stability of the material [6, 7].

The antioxidants commonly used in polymers are mainly divided into hindered phenols and phosphites. Hindered phenolic antioxidants, known as free radical scavengers, contain OH functional groups that can act as H donors to deactivate the free radicals (ROO•), stabilizing the material against photo- and thermal oxidation [8]. Phosphite antioxidants are auxiliary antioxidants, also known as hydroperoxides (ROOH) decomposers. When the hydroperoxides reach a certain concentration, the free radical oxidation reaction will be accelerated. Phosphite antioxidants can decompose the hydroperoxides produced in the oxidation process through changes in its phosphorus atom valence and generation of stable inactive products and play a role in stabilizing the polymer material.

Phosphite ester antioxidants generally have a synergistic effect when used together with hindered phenolic antioxidants to achieve the best antioxidant effect. The most frequently used hindered phenolic antioxidants are BHT, Irganox 3114 (3114), Irganox 1010 (1010), Irganox 1076 (1076), Irganox 1330 (1330), and so on [9–14]. The most frequently used phosphite antioxidant is Irgafos 168 (168). HPLC or HPLC-MS/MS is currently used to detect antioxidants. Schabron and Fenska have detected the antioxidants BHT, 1010, and 1076 in polyethylene by HPLC [15]. Moreta and Tena have detected the antioxidants 168, 1076, and 1010 in packaging plastics by LC-MS [16]. De Paepe et al. have analyzed antioxidants 1010, 1076, and 168 in polypropylene by supercritical fluid extraction [17]. These works only detect the contents of antioxidants in the extracts of packaging materials but neither develop the detection method for the determination of antioxidants transferred to the pharmaceutical solution from the packaging materials nor involve the detection method of degradation products of antioxidants. During the production and storage of pharmaceuticals, the antioxidants added to the packaging materials may change and degrade. The structure of antioxidant degradation products is similar to that of antioxidant, which has potential risks to human health safety. Therefore, it is necessary to detect the contents of antioxidants and their degradation products in the compatibility test of pharmaceutical solutions and their packaging materials.

**Figure 1:**
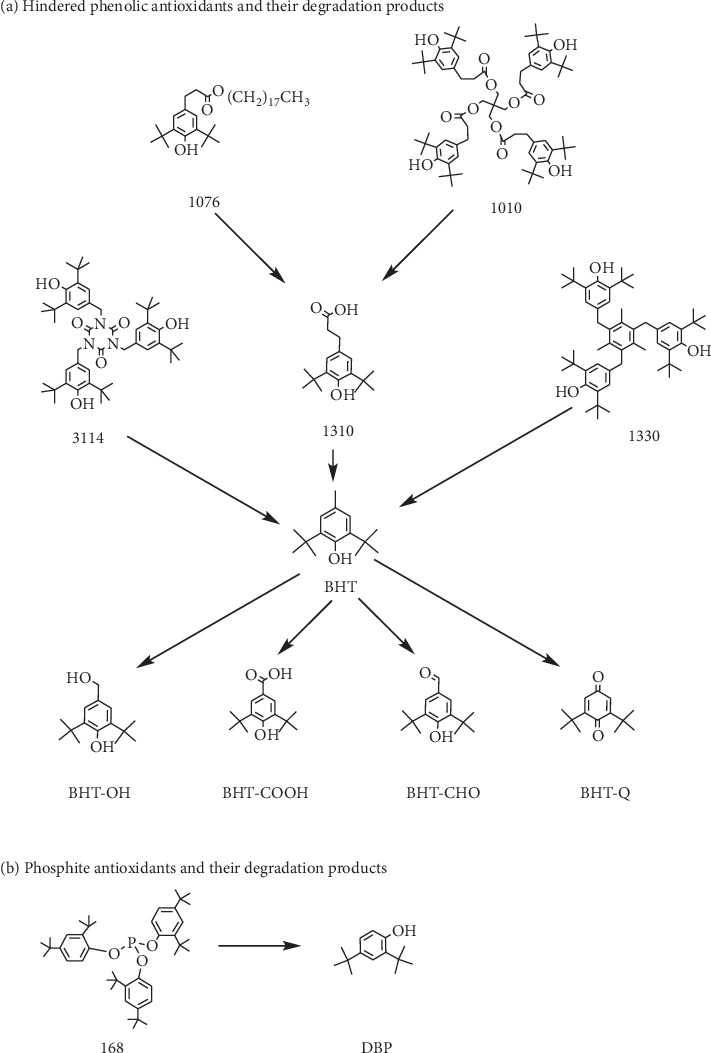
Schematic diagram of antioxidant degradation pathways. (a) Hindered phenolic antioxidants and their degradation products. The degradation products of BHT, 1310, 1330, and 3114 are mainly BHT-Q, BHT-OH, BHT-CHO, and BHT-COOH, respectively. (2) The degradation products of 1010 and 1076 are mainly 1310. (b) Phosphite antioxidants and their degradation products. The degradation product of 168 is mainly DBP.

The European Pharmacopoeia requires that the antioxidants BHT, 1010, 3114, and 1076 in polyolefin materials should be detected [18]. According to the European Pharmacopoeia and the formula information provided by manufacturers, the most commonly used antioxidants in the production of infusion bags are BHT, 3114, 1010, 1330, 1076, and 168. At present, the instruments used for quantitative analysis are mainly HPLC, HPLC-MS/MS, GC, and GC-MS/MS. The commonly used ion sources for quantitative analysis by HPLC-MS/MS are ESI source and APCI source, which have high sensitivity only for analytes with strong ionization ability. The ESI source has lower detection sensitivity for compounds with poor ionization capabilities, such as BHT and DBP, and the APCI source has lower detection sensitivity for compounds with higher boiling points, such as 1010, 3114, and 168. High-boiling compounds, such as 1010, 3114, and 168, cannot be detected by GC or GC-MS/MS. Therefore, HPLC-MS/MS, GC-MS/MS, or GC cannot simultaneously detect all antioxidants and their degradation products. Antioxidants and their degradation products contain aromatic groups, which are absorbed in the ultraviolet region. Currently, only the HPLC-UV method can detect antioxidants and their degradation products simultaneously. In the present study, we developed an HPLC method for the determination of antioxidants BHT, 3114, 1010, 1330, 1076, and 168 as well as their degradation products BHT-Q, BHT-OH, BHT-CHO, BHT-COOH, 1310, and DBP [19–22], which could simultaneously detect antioxidants and their degradation products rapidly and efficiently. In the extractable migration experiment, the solid-phase extraction (SPE) method was used to concentrate the analytes in the pharmaceutical solution to improve the detection sensitivity, which could evaluate the risk of antioxidant additives in infusion packaging materials migrating to the pharmaceutical solution. The relationship between antioxidants and their degradation products is shown in Figure 1.

## 2. Materials and Methods

### 2.1. Chemicals and Reagents

Methanol (HPLC grade) and acetonitrile (HPLC grade) were purchased from Fisher Scientific. Deionized water was produced with the Milli-Q® Advantage A10 water purification system (EMD Millipore Corporation). Dichloromethane was obtained from Chengdu Chron Chemicals Co,. Ltd. Acetic acid (HAc) was supplied by CNW Technologies. The sources and information of studied antioxidants and their degradation products are presented in Table 1.

### 2.2. Instruments and Optimized Chromatographic Conditions for Separation

Chromatographic separation of antioxidants and their degradation products was performed on the Waters e2695 Separation Modules equipped with a UV detector (monitoring at 277 nm) and an empower 2 data handling system (Waters Corporation, produced in the USA). The analytes were separated on a Waters SymmetryShield™ RP18 column (5 *μ*m, 250 × 4.6 mm) equipped with a security guard cartridge system (Phenomenex) at 35°C. The mobile phase A was acetonitrile, mobile phase B was methanol, and mobile phase C was HAc-water (1 : 99, v/v). The injection volume was 10 *μ*L. The HPLC system was operated at a flow rate 1.0 mL/min in a gradient mode. The analysis time was about 42 min. The gradient elution program of mobile phase is presented in Table 2.

### 2.3. Preparation of Standard and Sample Solutions

#### 2.3.1. Preparation of Standard Solutions

The standard stock solutions of individual antioxidants and their degradation products (BHT-OH, BHT-COOH, 1310, BHT-CHO, DBP, BHT, 3114, 1010, 1330, 1076, and 168) were prepared within a concentration range of 950–1,050 *μ*g/mL in mixture of dichloromethane and methanol (1 : 4, v/v). These solutions were mixed and diluted by methanol to prepare working standard solutions at a concentration of about 40 *μ*g/mL. The secondary standard solutions were diluted from working standards by methanol. The standard solutions were stored in amber-colored glass volumetric flasks at −20 °C.

#### 2.3.2. Extraction of Antioxidants and Their Degradation Products from Packaging Materials

The packaging materials were cut into small particles of about 5 mm × 5 mm, and then 0.5 g of packaging materials was weighed and placed in a microwave digestion tube, followed by an addition of 10 mL dichloromethane. The mixture was allowed to stand overnight (about 12 h), followed by microwave digestion at 40°C for 45 min and then cooled to room temperature. The extract solution was filtered through a glass funnel to remove solid particles and then vacuum-evaporated to remove the dichloromethane. The residue after evaporation was sonicated with 8 mL methanol. The solution was transferred to a 10 mL volumetric flask and diluted with methanol up to the mark. The solution was filtered with 0.45 *μ*m nylon membrane and analyzed by HPLC.

#### 2.3.3. SPE Procedure of Sodium Chloride Injection for Blood Transfusion Samples

The packaged 0.9% sodium chloride solutions for blood transfusion were stored for stability testing at 25 ± 2°C with RH of 40% ± 5% for 12 months, and sampling was performed at 0, 3, 6, and 12 months. SPE was used to concentrate the analytes to improve their detection sensitivity. Before sample loading, SPE cartridges (ChromcleanTM HLB 500 mg/6 mL, 30PK, SWEll) were conditioned with 3 mL methanol, followed by 10 mL deionized water. Subsequently, 50 mL sodium chloride (Nigale, China) was passed through the cartridges at a flow rate of 2 mL/min. The analytes were eluted into 10 mL brown glass volumetric flask using dichloromethane and methanol, and then the methanol was added to the mark of the volumetric flask.

### 2.4. Development of HPLC Method and Optimization of Chromatographic Conditions

The individual standard stock solutions (antioxidants and their degradation products) were diluted to about 5 *μ*g/mL with methanol, and each standard solution was, respectively, scanned by a UV-visible spectrophotometer (Evolution™ 201, Thermo Scientific) to detect the maximum absorption wavelength.

Mobile phase C consisting of deionized water and 0.1%, 0.2%, 0.5%, 1.0%, or 1.5% HAc was used to investigate the effects of different HAc contents in mobile phase C on the peak shape.

### 2.5. System Suitability Testing

System suitability testing is an integral part of liquid chromatography, which is used to verify whether the liquid chromatographic system is adequate for the intended quantitative analysis. United States Pharmacopeia (USP) requires testing for system suitability [23]. System suitability testing was performed by injecting working standard solutions at a concentration of about 40 *μ*g/mL in six replicates. The parameters, including repeatability, tailing factor, resolution, capacity factor, and theoretical plate, were studied.

### 2.6. Method Validation

The analytical method validation was evaluated by determining the following parameters: limits of detection and quantification (LOD and LOQ), linearity, precision, stability, and accuracy.

#### 2.6.1. LOD, LOQ, and Linearity

The stock solution containing 12 analytes (antioxidants and their degradation products) was diluted to a series of different concentrations and analyzed by HPLC. The calibration curves were constructed by plotting the peak areas versus the corresponding concentrations of each analyte. The linear range was 0–46.8 *μ*g/mL. The LOD and LOQ were calculated from the concentration of each group of analytes with a signal/noise ratio (S/N) at 3 and 10, respectively.

#### 2.6.2. Precision

Precision included intraday and interday precision, which was assessed by relative standard deviation (RSD) of the peak area of the spiked sample [24]. The mixed working standard solutions (250 *μ*L, 500 *μ*L, and 1,250 *μ*L) at a concentration of about 40 *μ*g/mL were added to the 50 mL sodium chloride solution. The spiked samples were processed according to the procedure in Section 2.3.3.

#### 2.6.3. Accuracy

Accuracy was assessed according to the recovery of the spiked sample. The mixed working standard solutions (200 *μ*L, 250 *μ*L, and 300 *μ*L) at a concentration of about 40 *μ*g/mL were added to 50 mL sodium chloride solution. The spiked samples were processed according to the procedure in Section 2.3.3.

#### 2.6.4. Stability

The stability was evaluated based on the RSD of peak area of the spiked samples. The mixed working standard solutions (250 *μ*L, 1,250 *μ*L, and 2,500 *μ*L) at a concentration of about 40 *μ*g/mL were added to 50 mL sodium chloride solution. The spiked samples were processed according to the procedure in Section 2.3.3 and analyzed at 0, 2, 4, 6, 8, 10, 12, 14, 16, 20, and 24 h.

## 3. Results and Discussion

### 3.1. Development of HPLC Method

**Figure 2:**
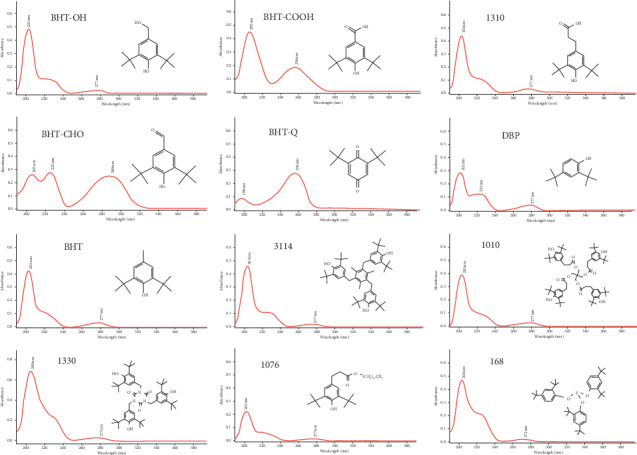
The UV spectra of antioxidants and their degradation products.

Antioxidants and their degradation products were aromatic compounds with two or three peaks in the UV absorption spectrum. The absorption peaks of antioxidants and their degradation products were about 200 nm close to the far ultraviolet region and 277 nm close to the visible region. Most organic compounds had absorption in the far ultraviolet region. In order to eliminate interference from other compounds during quantitative analysis, 277 nm, the maximum wavelength of the absorption peak close to the visible region, was selected as the wavelength of ultraviolet detection.  Figure 2 illustrates the corresponding ultraviolet absorption spectra of antioxidants and their degradation products.

The degradation products of antioxidants (BHT-OH, BHT-COOH, 1310, BHT-CHO, and DBP) contained hydrophilic groups, which had weak interaction with the C18 bonded phase on the chromatographic column and could be easily eluted. To improve the resolution of the degradation products of antioxidants, the proportion of water in the mobile phase was high in the first 19 min.

The solubility of antioxidants (BHT, 3114, 1010, 1330, 1076, and 168) in water was relatively low, which required methanol of a higher concentration to elute antioxidants from the chromatographic column. Antioxidants 1076 and 168 were more hydrophobic and had stronger interaction with the C18 bonded phase on the chromatographic column. In order to shorten the analysis time and improve the peak shape of 1076 and 168, the proportion of methanol was increased to 100% at the later stage of elution.

**Figure 3:**
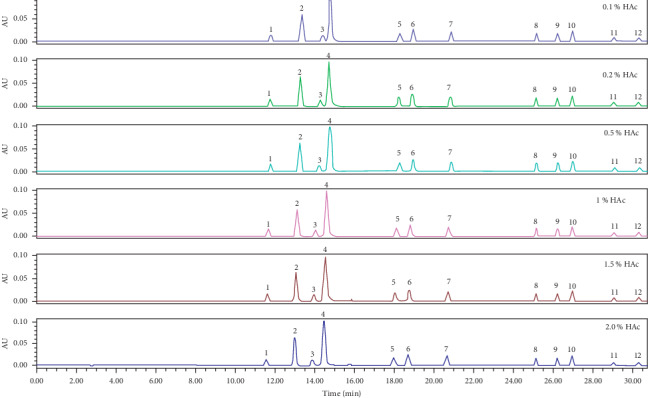
The peaks of antioxidants and their degradation products corresponding to different concentrations of HAc added to mobile phase C peaks: (1) BHT-OH, (2) BHT-COOH, (3) 1310, (4) BHT-CHO, (5) BHT-Q, (6) DBP, (7) BHT, (8) 3114, (9) 1010, (10) 1330, (11) 1076, and (12) 168.

In order to investigate the effect of mobile phase pH on peak shape and resolution, 0.1%, 0.2%, 0.5%, 1.0%, 1.5%, or 2.0% HAc was added to the deionized water (mobile phase C). When there was no HAc in the deionized water, most of BHT-COOH and 1310 were dissociated into their ions, and the binding capacity of BHT-COOH and 1310 with C18 column was weak, which was washed out earlier and with significant tailing. When HAc was added to the deionized water, the ionization of carboxyl groups of BHT-COOH and 1310 was inhibited. BHT-COOH and 1310 existed in the form of molecules and had a strong ability to bond with C18 column. Therefore, the retention time of BHT-COOH and 1310 was delayed. The acidic environment inhibited the ionization of silanols [25], and more than 99% of silanols were present in molecular state of Si-OH. Adding HAc to the mobile phase could inhibit the ionization of silanol group, prevent the tailing, and improve the resolution. The peaks of antioxidants and their degradation products corresponding to different concentrations of HAc in mobile phase C are shown in Figure 3.

**Figure 4:**
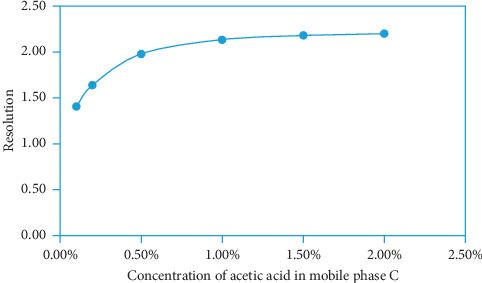
The resolution of 1310 between BHT-CHO corresponding to different concentrations of HAc in deionized water mobile phase C.

Mobile phase C consisted of HAc and deionized water. The resolution between 1310 and BHT-CHO was increased with the increase of HAc concentration in deionized water. When the concentration of HAc reached 1.0%, the resolution was >2.0. When the concentration of HAc was increased to 1.5%, the resolution changed little. Therefore, 1.0% HAc-water (1 : 99, v/v) was selected as the final concentration of HAc in the mobile phase C. The resolution of 1310 between BHT-CHO corresponding to different concentrations of HAc in mobile phase C is shown in Figure 4.

### 3.2. System Suitability Testing

**Figure 5:**
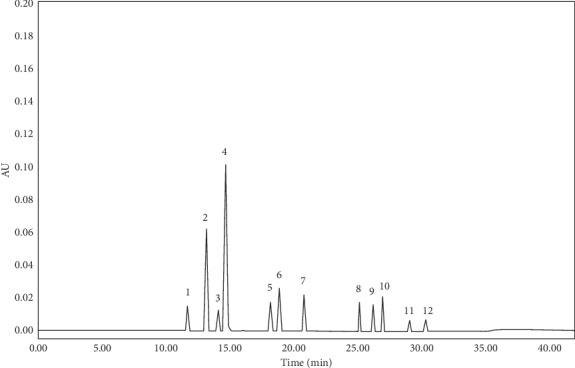
The typical HPLC chromatograms of 12 standard solutions under the optimized chromatographic conditions. The column temperature was 35°C (Waters SymmetryShield TM RP18 column, 5 *μ*m, 250 × 4.6 mm); the injection volume was 10 *μ*L; the UV detection wavelength was 277 nm; the flow rate was 1.0 mL/min; the mobile phase A was acetonitrile; mobile phase B was methanol; and mobile phase C was HAc-water (1 : 99, v/v). Peaks: (1) BHT-OH, (2) BHT-COOH, (3) 1310, (4) BHT-CHO, (5) BHT-Q, (6) DBP, (7) BHT, (8) 3114, (9) 1010, (10) 1330, (11) 1076, and (12) 168.

Table 3 shows the data of system suitability testing. The resolutions were >2; the average theoretical plates were >40,000; the capacity factor ranged from 10.72 to 29.35; the tailing factors ranged from 1.00 to 1.07; and the RSD of repeatability was <2.0%. The system suitability testing demonstrated that the HPLC system was adequate for the proposed analytical method.  Figure 5 shows the typical HPLC chromatograms of 12 standard solutions.

### 3.3. Method Validation

#### 3.3.1. LOD, LOQ, and Linearity

The LOD and LOQ were used to evaluate the sensitivity of analytical methods [26]. The LOD is the lowest concentration of analyte that can be detected. Generally, the LOD is the corresponding concentration of analyte when the signal-to-noise ratio is 3 : 1. The LOQ is the lowest concentration of analyte that can be quantified. Generally, the LOQ is the corresponding concentration of analyte when the signal-to-noise ratio is 10 : 1. The LOD and LOQ of the antioxidants and their degradation products were within the range of 0.011–0.151 *μ*g/mL and 0.031–0.393 *μ*g/mL, respectively.

Linearity is the degree of proportionality between the measured response value and analyte concentration. The linear correlations of the 12 analytes were good within the concentration range of about 0.1–40.0 *μ*g/mL and *r* > 0.999. The LOD, LOQ, and linearity of antioxidants and their degradation products are presented in Table 4.

#### 3.3.2. Precision

Precision refers to the closeness of agreement between the results obtained from multiple measurements of the same uniform sample under specified conditions. Precision was accessed by intraday precision and interday precision at three different concentration levels of antioxidants (1 *μ*g/mL, 2 *μ*g/mL, and 5 *μ*g/mL). Precision was evaluated by the RSD of the antioxidant peak area in three different days. The RSD range of intraday and interday precisions of analytes was 0.25–3.17% and 0.47–3.48%, respectively. The results showed good precision of the developed analytical method (Table 5).

#### 3.3.3. Accuracy

Accuracy is the degree of closeness between measurement results and the true or reference value [27]. Accuracy was accessed based on the recovery at three different concentration levels of analytes (0.8 *μ*g/mL, 1 *μ*g/mL, and 1.2 *μ*g/mL). The recoveries were obtained within the range of 80.39%–104.31%, indicating a good accuracy of the developed analytical method.  Table 6 lists the accuracy results.

#### 3.3.4. Stability

Stability was assessed by RSD of the analyte peak area in 24 h at three different concentration levels of analytes (1 *μ*g/mL, 2 *μ*g/mL, and 5 *μ*g/mL). The RSD of the peak area of each analyte was 0.35%–3.29%, indicating that there were no significant changes in each analyte in 24 h.  Table 7 shows the stability results.

### 3.4. Analysis of Samples

**Figure 6:**
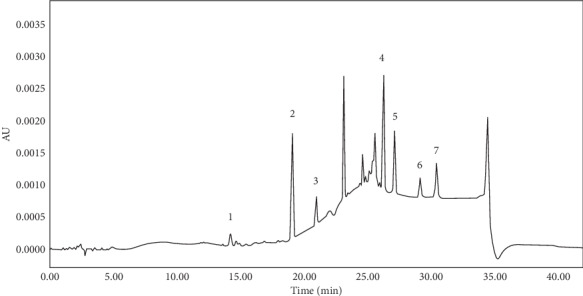
The HPLC chromatogram of the infusion bag extract. Peaks: (1) 1310, (2) DBP, (3) BHT, (4) 1010, (5) 1330, (6) 1076, and (7) 168.

**Figure 7:**
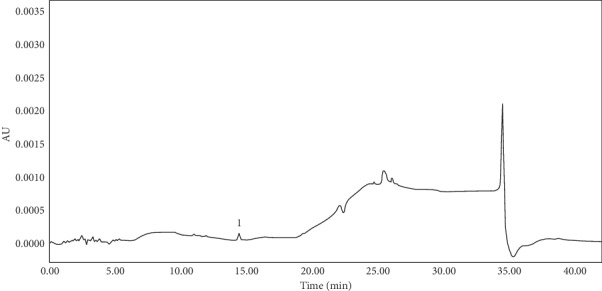
The HPLC chromatogram of extractables migrating into sodium chloride solution. Peak: (1) 1310.

The samples prepared in Sections 2.3.2 and 2.3.3 were determined by HPLC, and the peak area was substituted into the linear equation to calculate the sample concentration. Five antioxidants (BHT, 1010, 1076, 1330, and 168) and two antioxidant degradation products (1310 and DBP) were detected in packaging materials. In the extractable migration test, only 1310 was detected after the packaged pharmaceutical solution was stored for 0, 3, 6, and 12 months. Figures 6 and 7 illustrates the HPLC chromatograms of extractables in infusion bag and those migrating into the pharmaceutical solution, respectively.  Table 8 shows the permitted daily exposure (PDE) of antioxidants and their degradation products, the content of extracts in packaging materials, and the content of extractables that migrated into the pharmaceutical solution.

The PDE refers to the daily average maximum dose of a substance that is allowed to be ingested without any side effects to human body [28]. The maximum daily dose of sodium chloride injection for blood transfusion is about three bags (250 mL/bags). The results showed that the content of 1310 in samples at the time points of 0, 3, 6, and 12 months ranged from 37.44 *μ*g/3 bags to 48.71 *μ*g/3 bags, which was lower than the PDE of 1310 (300 *μ*g/day).

## 4. Conclusions

In the present work, five types of antioxidants and two types of antioxidant degradation products were detected in the infusion packaging materials. In the migration experiment, only 1310 was detected in the pharmaceutical solution. The antioxidants were not detected because antioxidants were almost insoluble in water, and they were difficult to migrate into the pharmaceutical solution.

The migrated amount of 1310 in the pharmaceutical solution at time points 0, 3, 6, and 12 months was low and barely changed. The content of 1310 in pharmaceutical solutions did not show a trend of sharp increase. Therefore, it would not cause drug safety risk. At present, most of the studies may not detect the degradation products of antioxidants because they have only developed the method to detect the antioxidants in the pharmaceutical solution. In the present work, 12 antioxidants and their degradation products were scanned by the ultraviolet spectrophotometer within the wavelength range of 190–400 nm, and the optimal detection wavelength for liquid phase conditions was determined to be 277 nm. The HPLC method for the determination of antioxidants (commonly used in the production of infusion bag packaging materials) and their degradation products was established, which could effectively separate 12 analytes in a short time. SPE was used to improve the sensitivity of analytes. The method was verified to be simple and reliable. Collectively, our method could be used not only for detecting antioxidants and their degradation products for infusion solution but also for other formulations.

## Figures and Tables

**Table 1:** Reagent information.

Abbreviation	CAS no	Chemical name	Source
BHT-OH	88-26-6	3,5-Di-tert-butyl-4-hydroxybenzyl alcohol	Alfa Aesar (China) Chemicals
BHT-COOH	1421-49-4	3,5-Di-tert-butyl-4-hydroxybenzoic acid	Alfa Aesar
1310	20170-32-5	3,5-Di-tert-butyl-4-hydroxyphenylpropionic acid	Alfa Aesar
BHT-CHO	1620-98-0	3,5-Di-tert-butyl-4-hydroxybenzaldehyde	Adamas Reagent Co., Ltd.
BHT-Q	719-22-2	2,6-Di-tert-butyl-p-benzoquinone	Adamas Reagent Co., Ltd.
DBP	96-76-4	2,4-Di-tert-butylphenol	TCI Company
BHT	128-37-0	2,6-Di-tert-butyl-4-methylphenol	Dr. EHrenstorfer GmbH
3114	27676-62-6	Tris(3,5-di-tert-butyl-4-hydroxybenzyl) isocyanurate	Aladdin
1010	6683-19-8	Pentaerythritol tetrakis(3-(3,5-di-tert-butyl-4-hydroxyphenyl)propionate)	Sigma-Aldrich
1330	1709-70-2	1,3,5-Trimethyl-2,4,6-tris(3,5-di-tert-butyl-4-hydroxybenzyl)benzene	Sigma-Aldrich
1076	2082-79-3	Octadecyl 3-(3,5-di-tert-butyl-4-hydroxyphenyl)propionate	Sigma-Aldrich
168	31570-04-4	Tris(2,4-ditert-butylphenyl) phosphite	TCI Company

**Table 2:** The gradient elution program of mobile phase.

Time (min)	Mobile phase A (acetonitrile, %)	Mobile phase B (methanol, %)	Mobile phase C (acetic acid-water, %)
0	10	50	40
3	10	50	40
6	20	50	30
9	25	50	25
12	25	50	25
15	30	50	20
19	0	90	10
21	0	100	0
31	0	100	0
32	10	50	40
42	10	50	40

**Table 3:** The result of system suitability testing (*n* = 6).

Analytes	RT (min)	Capacity factor, *K*′	Resolution, *R*	Tailing factor	Theoretical number of plate	Repeatability (RSD, %)
BHT-OH	11.72	10.72		1.07	44577	1.21
BHT-COOH	13.19	12.19	6.37	1.02	46945	0.53
1310	14.12	13.12	3.71	1.00	46948	0.83
BHT-CHO	14.68	13.68	2.11	1.06	43604	0.57
BHT-Q	18.20	17.20	13.06	1.01	77036	0.82
DBP	18.89	17.89	2.73	1.01	94260	0.45
BHT	20.83	19.83	8.30	1.00	139300	0.40
3114	25.16	24.16	22.21	1.01	351205	0.85
1010	26.23	25.23	5.77	1.02	261995	1.02
1330	26.98	25.98	3.52	1.02	231756	0.62
1076	29.09	28.09	8.54	1.03	181626	0.76
168	30.35	29.35	4.26	1.02	143418	0.43

**Table 4:** The LOD, LOQ, and linearity.

Analytes	LOD (*μ*g/mL)	LOQ (*μ*g/mL)	Calibration range (*μ*g/mL)	Linearity	*r*
BHT-OH	0.050	0.139	0.22～43.46	*y* = 5,930.9470 *x* + 1,825.7923	0.9996
BHT-COOH	0.015	0.058	0.11～45.28	*y* = 17,708.1278 *x* − 1,646.5349	1.0000
1310	0.057	0.238	0.49～39.14	*y* = 3,920.6502 *x* − 598.5409	1.0000
BHT-CHO	0.011	0.031	0.10～41.77	*y* = 33,938.8492 *x* − 5,298.7072	0.9999
BHT-Q	0.151	0.393	0.48～38.02	*y* = 2,292.7295 *x* − 1,549.6531	0.9994
DBP	0.042	0.116	0.19～38.38	*y* = 7,141.6319 *x* − 596.6704	1.0000
BHT	0.048	0.133	0.23～46.80	*y* = 5,549.7995 *x* − 577.9672	1.0000
3114	0.083	0.229	0.51～40.72	*y* = 3,338.2474 *x* − 524.0559	0.9999
1010	0.054	0.175	0.20～40.08	*y* = 3,775.4647 *x* − 298.1973	1.0000
1330	0.054	0.123	0.20～39.82	*y* = 5,433.0830 *x* − 415.6940	1.0000
1076	0.075	0.326	0.53～42.56	*y* = 2,093.7651 *x* − 303.7811	1.0000
168	0.122	0.371	0.52～41.71	*y* = 2,443.9582 *x* − 555.0495	1.0000

**Table 5:** The intraday and interday precisions (*n* = 9).

Analytes	Intraday precision (%)			Interday precision (%)		
	1 *μ*g/mL	2 *μ*g/mL	5 *μ*g/mL	1 *μ*g/mL	2 *μ*g/mL	5 *μ*g/mL
BHT-OH	1.31	0.64	2.00	1.37	1.66	1.43
BHT-COOH	0.99	0.82	1.76	1.24	0.96	1.31
1310	1.75	0.75	1.32	1.16	1.02	1.58
BHT-CHO	1.46	2.31	0.51	1.26	1.67	0.47
BHT-Q	0.67	0.30	3.17	2.21	2.78	3.48
DBP	1.52	0.27	2.11	1.27	1.94	1.48
BHT	3.08	3.03	1.91	1.80	2.41	1.32
3114	2.06	1.36	1.97	2.02	1.39	1.68
1010	3.04	1.01	2.26	1.96	1.82	1.70
1330	0.25	1.07	1.87	1.64	0.66	1.27
1076	0.30	0.72	0.73	0.83	0.56	0.79
168	1.89	0.71	0.53	1.41	0.67	0.54

**Table 6:** The results of accuracy (*n* = 3).

Analytes	0.8 *μ*g/mL		1.0 *μ*g/mL		1.2 *μ*g/mL	
	Recovery	RSD (%)	Recovery	RSD (%)	Recovery	RSD (%)
BHT-OH	94.84	1.46	93.99	4.24	91.54	1.94
BHT-COOH	102.31	0.38	102.42	0.48	101.49	0.98
1310	89.25	1.50	94.59	0.77	92.19	1.54
BHT-CHO	100.12	3.66	101.84	2.45	101.81	0.93
BHT-Q	88.11	1.99	87.68	2.95	80.39	3.42
DBP	104.31	0.70	103.68	0.90	103.87	0.92
BHT	103.37	1.28	102.43	0.48	102.41	0.49
3114	100.31	2.49	95.46	3.43	96.92	3.44
1010	103.65	1.12	103.00	1.13	102.59	1.88
1330	102.86	0.73	102.36	0.64	103.37	0.45
1076	95.14	2.03	98.07	1.82	96.07	3.42
168	98.17	0.83	98.19	0.45	99.61	4.20

**Table 7:** The stability of antioxidants and their degradation products in 24 h (*n* = 3).

Analytes	1 *μ*g/mL	5 *μ*g/mL	10 *μ*g/mL
	RSD (%)	RSD (%)	RSD (%)
BHT-OH	0.84	1.37	0.57
BHT-COOH	2.28	1.61	0.64
1310.00	1.09	3.29	1.33
BHT-CHO	1.27	1.51	0.43
BHT-Q	1.22	1.97	1.20
DBP	0.98	1.34	0.35
BHT	1.89	1.14	0.40
3114	0.91	1.24	0.47
1010	1.62	1.60	0.64
1330	1.13	1.27	0.40
1076	1.30	2.61	1.02
168	1.86	2.21	0.67

**Table 8:** Contents of antioxidants and their degradation products in samples.

Analytes	PDE (*μ*g/day)	Packing material extract (*μ*g/bag)	Extractables migrated into NaCl solutions for 6 months (*μ*g/bag)											
			0 month			3 months			6 months			12 months		
			Batch 1	Batch 2	Batch 3	Batch 1	Batch 2	Batch 3	Batch 1	Batch 2	Batch 3	Batch 1	Batch 2	Batch 3
BHT-OH	150	ND	ND	ND	ND	ND	ND	ND	ND	ND	ND	ND	ND	ND
BHT-COOH	150	ND	ND	ND	ND	ND	ND	ND	ND	ND	ND	ND	ND	ND
1310	300	383.04	46.51	39.43	41.69	38.03	37.44	43.12	38.32	43.48	40.83	48.71	42.65	48.34
BHT-CHO	150	ND	ND	ND	ND	ND	ND	ND	ND	ND	ND	ND	ND	ND
BHT-Q	150	ND	ND	ND	ND	ND	ND	ND	ND	ND	ND	ND	ND	ND
DBP	2500	2329.29	ND	ND	ND	ND	ND	ND	ND	ND	ND	ND	ND	ND
BHT	150	1832.25	ND	ND	ND	ND	ND	ND	ND	ND	ND	ND	ND	ND
3114	25000	ND	ND	ND	ND	ND	ND	ND	ND	ND	ND	ND	ND	ND
1010	10000	1825.23	ND	ND	ND	ND	ND	ND	ND	ND	ND	ND	ND	ND
1330	5000	443.13	ND	ND	ND	ND	ND	ND	ND	ND	ND	ND	ND	ND
1076	300	12287.52	ND	ND	ND	ND	ND	ND	ND	ND	ND	ND	ND	ND
168	2500	319.26	ND	ND	ND	ND	ND	ND	ND	ND	ND	ND	ND	ND

## Data Availability

All data included in this study are available upon request from the corresponding author.
